# Recent Progress in CFTR Interactome Mapping and Its Importance for Cystic Fibrosis

**DOI:** 10.3389/fphar.2017.00997

**Published:** 2018-01-17

**Authors:** Sang Hyun Lim, Elizabeth-Ann Legere, Jamie Snider, Igor Stagljar

**Affiliations:** ^1^Department of Biochemistry, University of Toronto, Toronto, ON, Canada; ^2^Department of Molecular Genetics, University of Toronto, Toronto, ON, Canada; ^3^Terrence Donnelly Centre for Cellular and Biomolecular Research, University of Toronto, Toronto, ON, Canada

**Keywords:** cystic fibrosis, CFTR interactome, protein-protein interactions, proteomics, interactome mapping

## Abstract

Cystic Fibrosis Transmembrane Conductance Regulator (CFTR) is a chloride channel found in secretory epithelia with a plethora of known interacting proteins. Mutations in the CFTR gene cause cystic fibrosis (CF), a disease that leads to progressive respiratory illness and other complications of phenotypic variance resulting from perturbations of this protein interaction network. Studying the collection of CFTR interacting proteins and the differences between the interactomes of mutant and wild type CFTR provides insight into the molecular machinery of the disease and highlights possible therapeutic targets. This mini review focuses on functional genomics and proteomics approaches used for systematic, high-throughput identification of CFTR-interacting proteins to provide comprehensive insight into CFTR regulation and function.

## Introduction

Cystic Fibrosis (CF) is a life-shortening genetic disease that affects multiple organs including the lungs, pancreas, liver, intestines, and the reproductive tract. The most prevalent symptoms, however, develop in the airways of CF patients with accretion of viscous mucus causing obstruction and an increased susceptibility to bacterial infection. Current treatment of CF is primarily symptom-based with the use of antibiotics to suppress bacterial colonization and physiotherapy to restore mucociliary clearance while enzyme supplements are given to patients who are pancreatic insufficient (Grasemann and Ratjen, 2010; Pasyk et al., 2017). Despite the recent approval of two new drug therapies (KALYDECO® and ORKAMBI®), which paved the way for personalized medicine for CF with a possible disease-modifying effect (Fajac and Wainwright, 2017), pulmonary function of CF patients progressively decreases due to recurrent bacterial infection and subsequent inflammation. In addition, the variation in survival and disease severity is extremely large and this heterogeneity is mostly attributed to variation in environmental factors and patients' genetic backgrounds.

CF is caused by mutations in the Cystic Fibrosis Transmembrane Conductance Regulator (CFTR) (Riordan et al., 1989) which is localized primarily in the apical membrane of secretory epithelial cells. The CFTR protein, regulated by phosphorylation and nucleotide binding, functions as an anion channel that mediates the flux of chloride and bicarbonate ions (Riordan, 2008), which provides the driving force for fluid transport. As its name suggests, CFTR also acts as a regulator influencing the activity of a variety of other channels and transporters (Li and Naren, 2010). Disease-causing mutations are associated with the loss of CFTR function at the surface of the airways, which leads to depletion of the airway surface fluid and accumulation of dehydrated and persistent mucus, the hallmark feature of CF (Boucher, 2007). There are currently over 2,000 mutations recorded, however the major mutation (F508del) is present in two-thirds of all CF patients. This mutation causes CFTR misfolding and subsequent retainment in the endoplasmic reticulum where it is targeted for degradation (Cheng et al., 1990). Interestingly, proper folding of the mutant proteins and their function can be partially restored by low temperature rescue (Denning et al., 1992), which reveals that post-translational processes play important roles in the manifestation of CF (Hutt et al., 2010). However, even upon rescue, mutant CFTR displays altered channel activity and reduced protein stability at the cell surface (Okiyoneda et al., 2010; Lukacs and Verkman, 2012), suggesting that there are many interacting proteins involved in proper folding, channel activity, and cell surface stability of CFTR (Riordan, 2005). Although the genetic cause of CF by CFTR mutations has been well studied in the past (Zielenski and Tsui, 1995), considerably less is known about the cellular protein environment of CFTR in CF.

## Identification of CF modifier genes

After the realization that the CFTR genotype alone could not account for all the phenotypic variation seen in the disease, linkage analysis and genetic association approaches found evidence of secondary factors affecting CF phenotypes, known as CF modifier genes (Kerem et al., 1990; Slieker et al., 2005; Weiler and Drumm, 2013). Since then, researchers have formed consortium studies and developed genome-wide association studies (GWAS) to elucidate candidate genes in many aspects of CF such as lung function, meconium ileus, and CF related diabetes (Drumm et al., 2005; Wright et al., 2011; Sun et al., 2012; Blackman et al., 2013). As a result, the impact of secondary, non-CFTR genes in modifying the severity of the CF phenotype has gained a growing importance in the field and has been investigated by several groups (Cutting, 2010; Dorfman, 2012). For the most part, the biological roles of these modifier genes in affecting CF phenotypes are not well documented, but, to date, several have been linked to regulation of the inflammatory response (Gu et al., 2009a), protein folding and degradation (Emond et al., 2012), apoptosis (Wright et al., 2011), or ion transport (Sun et al., 2012).

One example of a well-known CF gene modifier is EHF (ETS Homologous Factor), involved in the inflammatory response. The regulatory intergenic region of EHF and APIP (APAF1 Interacting Protein) (11p13) was found to contain a single-nucleotide polymorphism (SNP) that had a strong association with lung function in F508del homozygotes (Wright et al., 2011). This finding was confirmed in three separate studies; these included an additional 2,900 patient genome sequences (Corvol et al., 2015), targeted resequencing analysis (Dang et al., 2016) and a more recent functional follow-up (Stanke et al., 2014). Although less characterized, other SNPS found to be associated with lung function in CF are in areas defined by the genes MUC4, MUC20, HLA Class 11, AGTR2, SLC9A3 (Corvol et al., 2015), SLC26A9, SLC6A14 (Sun et al., 2012), and IFRD1 (Gu et al., 2009a).

Being part of the CFTR genetic interaction network, these novel variants and modifiers help explain why there is a large phenotypic variation in CF patients. In addition, modifier genes may represent possible therapeutic targets for CF treatment in follow-up studies (Cutting, 2010; Yi et al., 2013). However, as interesting as it is to find them, the detailed mechanism by which many of the identified genes affect the severity of CF remains elusive, and whether they physically and/or functionally associate with CFTR itself is still not completely understood (Gu et al., 2009b; Stolzenburg et al., 2017). In order to design improved therapies for treatment of CF, and better understand the mechanism of action by which these therapies work, it is important to obtain a comprehensive knowledge of the proteins associated with both wild type CFTR and the mutated CFTR variants responsible for CF.

## Importance of studying CFTR-interacting proteins

Protein-protein interactions (PPIs) carry out many cellular processes with temporal and spatial precision (Eckford and Bear, 2011). Their inherent dynamic nature allows cells to adjust in response to stimuli and environmental conditions with flexibility in function (Snider et al., 2015). Therefore, dysfunction of PPIs can have systemic consequences resulting from the perturbation of the interconnected cellular networks (Barabási et al., 2011) leading to disease phenotypes such as those seen in CF.

Not surprisingly, CFTR interacts with a wide variety of proteins that play a major role in various aspects of CF (Riordan, 2005; Li and Naren, 2010). For instance, the phosphorylation and localization of CFTR to the apical membrane, which is crucial for its regulation and proper function, requires a complex PPI network mediated via PDZ interactions (Guggino, 2004; Li and Naren, 2010). CFTR is also known to regulate the activities of other transporters and channels, hence PPIs that can affect the expression or the function of the CFTR channel at the plasma membrane can be of broad physiological significance as well. Furthermore, it has been speculated that cellular background plays an important role in the ability of F508del CFTR to be rescued from degradation (Wang et al., 2008), with targeting of molecular chaperones as a potential therapeutic approach. The importance of PPIs involved in CF is further strengthened as recent clinical studies that tested CFTR-modulating small molecules have only shown modest effects (Wainwright et al., 2015).

As a result, there has been a growing interest in CFTR proteomics over the last decade, with analysis of CFTR-interacting proteins becoming an important means of understanding the cell-specific environments associated with wild type and mutant CFTR, and their corresponding functional consequences (Wang and Li, 2001; Li and Naren, 2005; Collawn et al., 2010).

## Methodological overview

So far, the approaches used to map protein interactomes can be divided into three classes (Vidal et al., 2011). The first involves combining experimentally validated PPIs from multiple small-scale studies. The second involves computational prediction algorithms, such as the recently developed FpClass (Kotlyar et al., 2014), which make use of a variety of available known datasets and protein features to generate lists of predicted potential interactions. And finally, the third employs systematic high-throughput experimental mapping strategies. Notably, mapping systematic and comprehensive interaction networks was challenging due to low accuracy during early implementations (von Mering et al., 2002). However, advances in data analysis and methods to empirically assess protein interaction mapping quality have enabled robust validation of the accuracy and sensitivity of datasets acquired using high-throughput approaches, something which isn't possible with the results of small-scale experiments used for curated databases (Venkatesan et al., 2009; De Las Rivas and Fontanillo, 2010; Rolland et al., 2014). There are two general types of methodologies widely used for large-scale PPI mapping; variations of two-hybrid/protein complementation systems and protein purification techniques followed by mass spectrometry (De Las Rivas and Fontanillo, 2010). Two-hybrid/protein complementation datasets mostly contain binary interactions while mass spectrometry datasets contain a mix of both direct associations and indirect interactions that may occur in the context of protein complexes.

Elucidation of protein interactors of CFTR using systematic strategies has traditionally been difficult due to the complex biochemical features of this integral membrane protein. Many of the protein purification-based techniques employed are not high throughput-compatible and often require optimization for each protein complex examined. Additional technical constraints such as the low endogenous expression of CFTR and the lack of high-quality anti-CFTR antibodies also present complications and often demand use of epitope-tagged CFTR. Despite these difficulties, assays such as GST-pull downs, co-immunoprecipitation, and affinity purification-mass spectrometry (AP-MS) have been used to elucidate potential CFTR-interacting proteins (Sun et al., 2000a; Cheng et al., 2002; Scroggins et al., 2007). Genetic methods such as the yeast two hybrid have also been used, albeit with just the cytosolic domains of CFTR, to identify interactors (Raghuram et al., 2002; Kim et al., 2004; Duan et al., 2012). However, our knowledge of the comprehensive human CFTR interactome is still at an early stage, as only a few studies have examined CFTR interactions on a global scale (Wang et al., 2006; Pankow et al., 2016).

## Current knowledge of the global CFTR interactome

**Figure 1:**
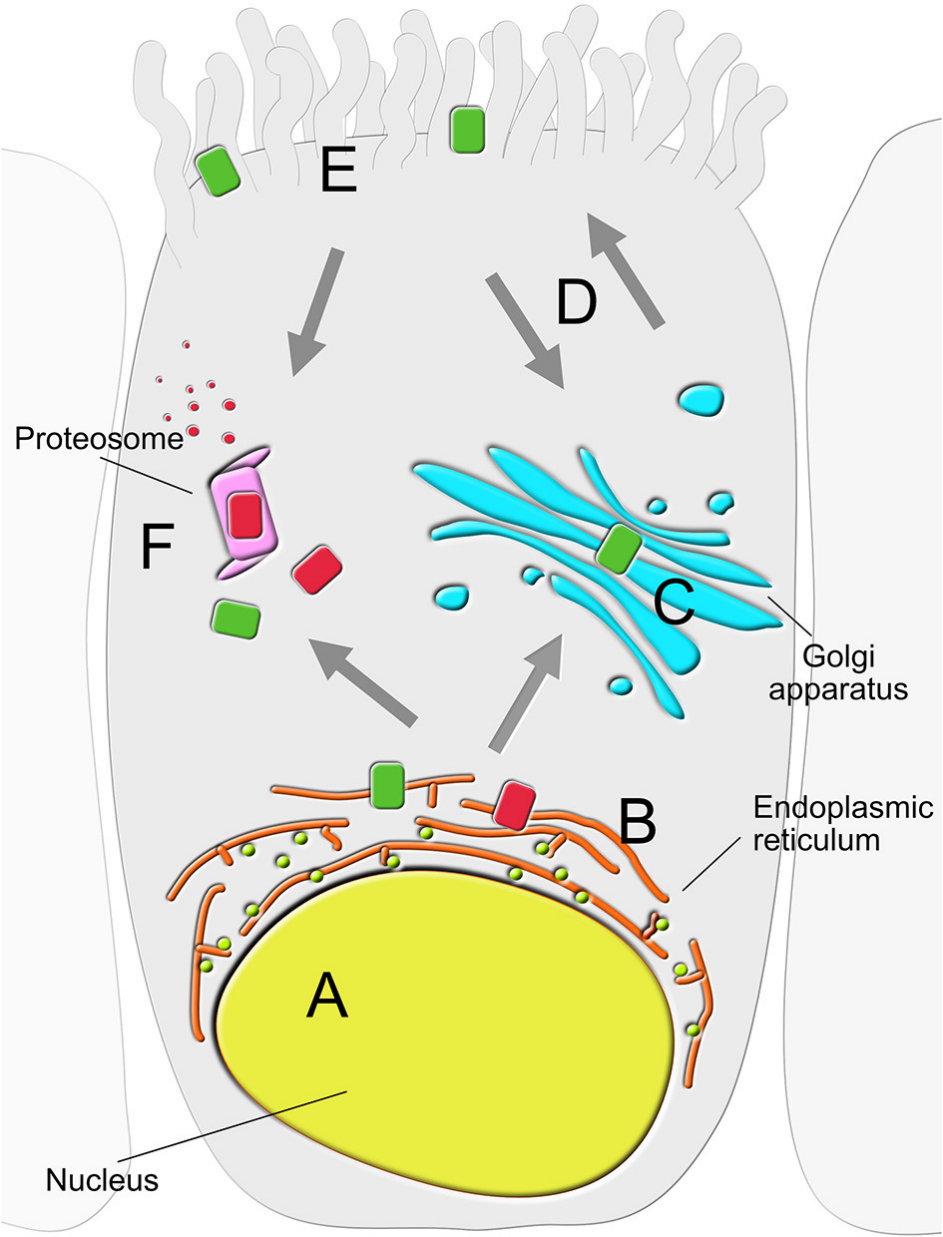
Location of CFTR-interacting proteins in the secretory epithelial cell. Physical and functional interactors of wild type (green) and mutant (red) CFTR can be largely grouped into six clusters based on the primary location where the interaction takes place; **(A)** nucleus, **(B)** endoplasmic reticulum (ER), **(C)** Golgi apparatus, **(D)** trafficking vesicles, **(E)** plasma membrane (PM) and **(F)** proteasome.

CFTR-interacting proteins identified to date can be broadly categorized based on the cellular location where the interaction takes place (Figure 1). These include the nucleus (Sood et al., 1992), ER (Wang et al., 2006; Chanoux and Rubenstein, 2012; Pankow et al., 2015), proteasome (including ER-associated degradation pathway; Ameen et al., 2007; Chanoux and Rubenstein, 2012; Lopes-Pacheco et al., 2015; Pankow et al., 2015), Golgi apparatus (Zhang et al., 2002; Guggino and Stanton, 2006), membrane trafficking vesicles (Wang et al., 2006; Ameen et al., 2007; Chanoux and Rubenstein, 2012), plasma membrane (Sun et al., 2000b; Klein et al., 2016; Bertrand et al., 2017), and the cytoskeleton (Monterisi et al., 2012; Edelman, 2014). Below we discuss key findings from some of the large-scale approaches applied to comprehensively investigate the biological significance of PPIs in CF. These were designed to map dynamic interactomes with expectations of discovering novel proteins associated with human CFTR, and to uncover new biochemical pathways that, when defective, contribute to CF.

## Mass spectrometry results

Mass spectrometry has become a popular method for characterizing the protein components of biological systems as it provides a framework for gaining insights into the composition of molecular complexes with possible regulation mechanism and function (Cravatt et al., 2007). One early report of using large-scale proteomics to understand global CFTR protein interactions came from Wang et al. (2006), in which they applied mass spectrometry through use of Multidimensional Protein-Identification Technology (MudPIT) (Wolters et al., 2001) to analyze chaperone assemblies that regulate CFTR folding and transport (Wang et al., 2006). The number of CFTR-associated proteins, including known CFTR-binding chaperones and unknown interactors, identified in this study was unprecedented. Three Hsp90 co-chaperones were found to be involved in regulating channel folding and/or export from the ER, and by using RNA-interference (RNAi) knockdown, Aha1 was discovered to inhibit coupling of F508del CFTR to the ER export machinery (Wang et al., 2006). The authors discovered that a reduced level of Aha1 alters the Hsp90-CFTR interaction by promoting the transition from folding to the export pathways. This study introduced the concept of the CFTR “chaperome,” a dynamic cellular chaperone folding environment that can be subjected to pharmacological intervention.

Later, the group moved on to establish comprehensive dynamic interactomes for both wild type and F508del CFTR using an improved purification technology termed Co-interacting Protein Identification Technology (CoPIT) (Pankow et al., 2015). They found 638 high-confidence interactors that further enriched the previously existing core CFTR interactome. Substantial insight into the molecular mechanisms of CF was obtained by analyzing the extensively remodeled F508del CFTR interactome upon rescue. Until then, there were few proteins whose known interaction with CFTR involved mutant stabilization and partial restoration of channel activity. However, in this study, Pankow et al. (2015) identified 209 proteins that differ significantly in the relative amounts recovered between the F508del and wild type CFTR cell lines, representing highly enriched wt/mutant specific interactors. In addition, the F508del CFTR-specific interactome was characterized mainly by gain of novel interaction partners, which revealed distinct differences in the biogenesis of the two CFTR proteins. Not surprisingly, recruitment of chaperones and protein degradation mediators of ER quality control network was found to be enhanced in the F508del CFTR-specific interactome. Also, PPIs involved in translation, post-translational modification, protein transport and trafficking, and endocytic recycling were altered, suggesting that large aspects of CFTR biogenesis are affected by deletion of F508 (Pankow et al., 2015). Notably, the F508del CFTR interactome dynamics under a lower temperature rescue condition resulted in reduced interactions with proteins involved in ER quality control. The authors also investigated interactome remodeling upon HDACi and validated their key interactors by performing an RNA interference screen. This work from Pankow et al. is the most exhaustive proteomics project involving human CFTR performed to date reporting that the loss of F508del CFTR function involves novel associations with protein complexes and cellular pathways that differ compared to wild type (Pankow et al., 2015).

The CoPIT method has been further utilized in the most recent work by Reilly et al. (2017) to identify a novel role of the PI3K/Akt/mTOR pathway in CF (Reilly et al., 2017). They demonstrated that inhibiting the upregulated mTOR activity in F508del CF bronchial epithelial cells resulted in increased CFTR stability and expression.

## Two-dimensional gel electrophoresis (2-DE) approaches

Traditional proteomics methodologies involved separation of protein samples by isoelectric point and molecular weight, with the addition of mass spectrometry analysis in more recent years. This proteomic approach was first applied to the identification of protein characteristics of CF by Pollard et al. (2005) using CF cell lines IB3-1 and its wild-type CFTR-repaired daughter cell line CFTR IB3-1/S9 (Pollard et al., 2005). They identified 194 proteins including a high abundance of chaperones, proteasome proteins, and calcium signaling and pro-inflammatory proteins associated with the NFκB pathway, serving as a foundation reference database for proteomic studies on CF cells. Involvement of keratins in transferring CFTR to the plasma membrane was identified through this method by Davezac et al. (2004). Using 2-DE and Western blot approaches, another group identified proteins involved in unfolded protein response (UPR) and cell metabolism that promote low temperature-induced F508del CFTR rescue (Gomes-Alves et al., 2009). The same group later discovered that proteome modulation associated with the UPR contributes to the rescue of F508del CFTR by RXR motif inactivation (Gomes-Alves et al., 2010).

Identification of a protein with different binding affinity to the mutant form of CFTR than to CFTR wild type was also discovered using a 2-DE method. Teng et al. (2012) identified calumenin as a new CFTR chaperone that is present in higher abundance with a complex containing G551D CFTR, which is another CF-causing missense mutation resulting in altered channel activation (Teng et al., 2012). They first resolved proteins co-immunoprecipitated with G551D CFTR by 2-DE then applied mass spectrometry to spots that were different in intensity compared with the wild type distribution. By using co-immunoprecipitation, the authors were able to determine that though the basal expression of calumenin is similar between G551D and wild type CFTR-expressing cells, the amount of calumenin bound to the complex in G551D CFTR cells is higher with increased expression of Grp78, a protein involved in the UPR.

Singh et al. (2006) applied the chemical chaperone 4-Phenylbutyrate (4-PBA), an oral butyrate derivative used for treatment of urea cycle disorders, for their investigation of molecular networks involved in promoting CFTR processing. Proteome profiling of bronchial epithelial cells treated with the compound revealed 85 differentially expressed proteins that became part of the first pharmaco-proteomics map of CFTR (Singh et al., 2006). Later the authors further characterized their findings and concluded that the interactome of CFTR channel rescued by 4-PBA contains a set of HSP70 family proteins that constitute potential therapeutic networks for targeted intervention (Singh et al., 2008).

## Functional genomics approaches

While the above studies examined the interactomes and expression patterns associated with various forms of CFTR using proteomics (Collawn et al., 2010), others have used a high-content screen based on functional assays (Trzcinska-Daneluti et al., 2009). Using a high-throughput imaging system and co-expression of proteins fused to a halide-sensitive yellow fluorescent protein (YFP), Trzcinska-Daneluti et al. (2009) identified 13 proteins whose overexpression enhanced F508del CFTR rescue in both HEK293 and BHK cells. Of those, they emphasized STAT1, finding that F508del CFTR can also be rescued by knocking down PIAS1, which is a known protein inhibitor of activated STAT1. They later applied this assay with RNAi screens to identify novel suppressors of F508del CFTR maturation and discovered that inhibition of FGFR signaling by chemical compounds leads to altered chaperone expression and robust rescue of F508del mutant (Trzcinska-Daneluti et al., 2015).

Similarly, another group used a method which involved siRNA gene knock-down coupled with high content microscopy for readouts. Botelho et al. (2015) developed a plasma membrane protein traffic assay in the CF Bronchial Epithelial (CFBE) cell line that captures traffic efficiency of CFTR protein using double-tagged reporter and high-throughput (HT) microscopy (Botelho et al., 2015). Although their method is mainly reported as a new platform for HT screening drug discovery, combination with a small-scale siRNA screen enabled the authors to identify COPB1 and OR2AG1 as novel CFTR therapeutic target genes. This platform has the potential to be applied in larger scale to identify novel CFTR traffic regulators.

Putative protein targets that have been shown to influence CFTR biogenesis and function in CF using systemic proteomic approaches are listed in Table 1.

**Table 1:** Putative CFTR-interacting targets for restoring CFTR biogenesis and function in CF identified through proteomic approaches.

**Targets**	**Function**	**Interacting CFTR variant**	**Location**	**Effects on CFTR biogenesis/function**	**Ref**.
Aha1	Hsp90 cochaperone ATPase regulator	F508del	ER	Downregulates the expression and maturation of F508del CFTR	Wang et al., 2006
PABPC1	RNA processing and co-translational control		Cytosol	Knockdown promotes F508del CFTR maturation and enhances forskolin/genistein-stimulated F508del CFTR channel activity in primary CF epithelia	Pankow et al., 2015
PTBP1			Nucleus		
YBX1			Nucleus/Cytosol		
TRIM21	E3 ubiquitin-protein ligase; proteosomal degradation		Cytosol		
PDIA4	Potential novel components of ER quality control		ER		
SURF4			ER/Golgi		
PTPLAD1			ER		
LGALS3BP	Lectin Galactoside-Binding Soluble 3-Binding Protein		Extracellular/PM	Knockdown results in loss of expression and channel activity of F508del CFTR	
BAG3	Bcl-2-associated athanogene 3; co-chaperone of Hsp70/Hsc70		Nucleus/Cytosol	Inhibition reduces F508del CFTR aggregates	Reilly et al., 2017
KRT18	Type I intermediate filament chain Keratin 18		Cytoskeleton	Redistribution and/or decreasing the expression promotes F508del CFTR delivery to the plasma membrane	Davezac et al., 2004
Psme2	Proteasome activator 28 subunit beta (PA28β)	F508del & F508del/4RK-CFTR	Proteasome	Differential expression pattern observed with low temperature treatment for F508del CFTR rescue in BHK cells	Gomes-Alves et al., 2009, 2010
Cops5	COP9 Signalosome Subunit 5		Cytosol		
RACK1	Receptor For Activated C Kinase 1		ER/vesicles/PM		
Calumenin	Calcium-binding protein involved in protein folding and sorting	G551D	ER	Increased level of interaction with G551D CFTR during maturation/trafficking compared to wild type CFTR	Teng et al., 2012
GRP94 (HSP90B1)	Heat Shock Protein families	F508del	ER	Association with immature CFTR upon 4-PBA modulation	Singh et al., 2006, 2008
HSP84 (HSP90AB1)					
GRP78 (HSPA5)					
GRP58 (PDIA4)	Protein Disulfide Isomerase Family A Member 3				
STAT1	Signal transducer and activator of transcription 1		Cytosol/Nucleus	Rescues F508del CFTR function better than Corr-4a treatment when co-expressed	Trzcinska-Daneluti et al., 2009
FGFR1	Fibroblast Growth Factor Receptor 1		ER/vesicles/PM	Inhibition of FGFR signaling pathway upregulates F508del CFTR maturation	Trzcinska-Daneluti et al., 2015
COPB1	Coatomer Protein Complex Subunit Beta 1	Wild type	Vesicles/Golgi	Presumed wild type CFTR traffic inhibitor	Botelho et al., 2015
OR2AG1	Olfactory Receptor Family 2 Subfamily AG Member 1		PM	Presumed wild type CFTR traffic enhancer	
USP19	Ubiquitin Specific Peptidase 19	F508del	ER/Cytosol/PM	Rescues F508del CFTR when overexpressed	Hassink et al., 2009
NYD-SP27 (PLCZ1)	Phospholipase C isoform		Cytosol	Suppression results in increased F508del CFTR trafficking and restoration of pancreatic anion secretion	Zhu et al., 2003
HDAC7	Histone deacetylase 7		Nucleus/Cytosol	Chemical inhibition or siRNA knockdown restores F508del CFTR function	Hutt et al., 2010

## Lack of protein complementation assay

Although both protein complementation systems and purification techniques followed by mass-spectrometry are widely used methods for large-scale mapping of PPI, with respect to proteomic profiling of CFTR, our current knowledge is mainly based on data acquired via mass spectrometry approaches. This is due to the fact that two-hybrid/protein complementation systems are limited by technical challenges associated with the high-throughput study of full-length integral membrane proteins in human cells. For example, a significant limitation of the traditional two-hybrid screening system is the host organism, yeast, which does not have the same post-translational modification machinery as mammalian cells and has different membrane composition that are not ideal for studying human receptors and transporters whose protein networks dynamically change in response to ligand binding, drug inhibition and changes in microenvironment. Also, traditional two-hybrid requires the use of only the soluble domain of membrane proteins, rather than their proper full-length form, and can consequently lead to missing or spurious interaction. These factors have limited our ability to detect PPIs between human CFTR and its interacting partners using two-hybrid screening systems on a global scale. To address these problems, new proteomics screening technologies for use in mammalian cells are being developed such as the Mammalian Membrane Two-Hybrid (MaMTH) (Petschnigg et al., 2014, 2017; Saraon et al., 2017; Yao et al., 2017). Future large-scale studies using these new protein complementation assays should therefore detect additional binary CFTR interactors that will certainly complement the current mass spectrometry datasets.

## Concluding remarks

It is evident that phenotypic manifestation of CF is intricately connected with altered PPIs that are part of the proteostasis network (Amaral and Balch, 2015). Therefore, studying the differences in cellular protein expression and interaction profiles caused by the loss of CFTR or expression of mutants using functional genomics and proteomics is important for identifying novel therapeutic intervention approaches and developing new diagnostic tools. Indeed, proteomics approaches are beginning to provide immense information on the physiological function of CFTR and the environment in which it functions. This mini review has examined some of the currently available methods employed and the work dedicated to characterizing proteins associated with CFTR. The growing CFTR interactome will serve as a powerful resource for the CF community, and will be invaluable in furthering our understanding of CFTR function and in the identification of novel CF therapeutic targets. Merging genomic, proteomic, and other functional technologies will lead to a paradigm shift in CF health care and will be a step forward in personalized management of the disease (Amaral and Balch, 2015).

## Author contributions

SL wrote the bulk of the manuscript and produced the figure. EA-L was involved in the writing of the manuscript and referencing. JS was involved in the editing and critical review of the manuscript. IS designed the overall layout and critically reviewed the manuscript.

### Conflict of interest statement

The authors declare that the research was conducted in the absence of any commercial or financial relationships that could be construed as a potential conflict of interest.

## Abbreviations

APIP: APAF1 Interacting Protein
CF: Cystic Fibrosis
CFTR: Cystic Fibrosis Transmembrane Conductance Regulator
EHF: ETS Homologous Factor
PPIs: Protein-Protein Interactions
SNP: Single-Nucleotide Polymorphism.
